# Bayesian alternatives for common null-hypothesis significance tests in psychiatry: a non-technical guide using JASP

**DOI:** 10.1186/s12888-018-1761-4

**Published:** 2018-06-07

**Authors:** Daniel S. Quintana, Donald R. Williams

**Affiliations:** 1 NORMENT, KG Jebsen Centre for Psychosis Research, Division of Mental Health and Addiction, University of Oslo, and Oslo University Hospital, Building 49, Oslo University Hospital, Ullevål, Kirkeveien 166, PO Box 4956, N- 0424 Nydalen, Oslo Norway; 2 Department of Psychology, University of California, Davis, Davis, CA USA

**Keywords:** Statistics, Bayesian analysis, Research methods, *p*-values, Null hypothesis significance testing, Software

## Abstract

**Background:**

Despite its popularity as an inferential framework, classical null hypothesis significance testing (NHST) has several restrictions. Bayesian analysis can be used to complement NHST, however, this approach has been underutilized largely due to a dearth of accessible software options. JASP is a recently developed open-source statistical package that facilitates both Bayesian and NHST analysis using a graphical interface. This article provides an applied introduction to Bayesian inference with Bayes factors using JASP.

**Methods:**

We use JASP to compare and contrast Bayesian alternatives for several common classical null hypothesis significance tests: correlations, frequency distributions, t-tests, ANCOVAs, and ANOVAs. These examples are also used to illustrate the strengths and limitations of both NHST and Bayesian hypothesis testing.

**Results:**

A comparison of NHST and Bayesian inferential frameworks demonstrates that Bayes factors can complement *p*-values by providing additional information for hypothesis testing. Namely, Bayes factors can quantify relative evidence for both alternative and null hypotheses. Moreover, the magnitude of this evidence can be presented as an easy-to-interpret odds ratio.

**Conclusions:**

While Bayesian analysis is by no means a new method, this type of statistical inference has been largely inaccessible for most psychiatry researchers. JASP provides a straightforward means of performing reproducible Bayesian hypothesis tests using a graphical “point and click” environment that will be familiar to researchers conversant with other graphical statistical packages, such as SPSS.

## Background

The prevailing inferential framework for summarizing evidence in psychiatry is null hypothesis significance testing (NHST), which is a hybrid of Fisherian and Neyman-Pearson statistics [1]. NHST generates a test-statistic, such as a *t*-value, and then the probability (*p*-value) of observing this value or a more extreme result is computed, assuming that the null hypothesis is true. *P*-values are used in concert with alpha and beta levels to minimize false-positive (Type I) and false-negative (Type II) errors in the long run by either rejecting or failing to reject the null hypothesis. If interpreted as a measure of discrepancy from a null sampling distribution, *p*-values can be especially informative [2].

Despite its enduring popularity, the *p*-value has been the subject of a growing chorus of criticism. Excellent treatments of *p*-value limitations and common misunderstandings are already available [3, 4], so we will only briefly cover two issues especially relevant for psychiatry research. First, as the traditional *p*-value approach is only concerned with disproving the null hypothesis, there is no way to assess if the data favors the null hypothesis compared to the alternative hypothesis. Even a “large” non-significant *p*-value does not provide evidence for the null hypothesis [5]. Consequently, examining statistical equivalency is beyond the reach of conventional *p*-value test approaches — but see the “two one-sided test” for an approach that uses the same framework underlying *p*-values [6, 7]. Second, unless an a priori power analysis is performed, there is no clear indication if a dataset is sensitive enough to detect a true effect when using *p*-values [8].

*P*-values and alpha levels fall under the classical school of frequentist statistics, and are used to control long-run error rates. The Bayesian framework [9, 10] offers an alternative approach, as it allows for the probabilistic description of parameters and hypotheses. There have been several publications detailing the philosophical and practical differences between these two viewpoints [3, 11, 12], but it suffices for our purposes to note that only the Bayesian framework allows us to quantify how much more likely the data are under the null hypothesis (*H*_0_) compared to the alternative hypothesis (*H*_1_), given a prior probability. A Bayes factor, which is a popular implementation of Bayesian hypothesis testing, can quantify the degree to which the data favor one of two hypotheses by considering the prior odds. It is important to note that the Bayesian framework also includes parameter estimation, which can address the size of an effect [for an excellent treatment of Bayesian parameter estimation, see [10]]. While Bayesian parameter estimation is a valuable tool, hypothesis testing via Bayesian model comparison can facilitate theory prediction by providing a measure of relative evidence between two models [13], typically a null and alternative model.

Specifying a prior distribution of the parameter in a statistical model is central to Bayesian inference, and serves many purposes such as improved parameter estimation [14–16]. We will return to prior distributions in the examples below, but will now provide a brief summary. A prior distribution can quantify, or at least approximate an idealized concept of, prior information about the parameters of the model *before* the data is considered. Unlike classical inferential frameworks, Bayesian inference can incorporate such prior knowledge [17]. For instance, if dealing with an effect size parameter, such as Cohen’s *d*, we may judge a priori that values of *d* less than − 1 or greater 1, are much less likely than *d* values around 0. This is a fair assumption for biobehavioral research, which tends to yield small-to-medium effect sizes (i.e., *d =* 0.2 to *d* = 0.5). If a parameter is unconstrained, the use of a Cauchy distribution centered around an effect is a common approach [18]. This distribution is typically centered on zero by default, but can be also centered elsewhere. The Cauchy distribution is similar to a normal distribution, but has fatter tails and less central mass [19]. Normal and *t*-distributions are also common choices for prior distributions [20]. It should be noted that the lack of general rules for choosing priors is often used as an objection against the Bayesian framework. Uniform default priors, which suggest that any parameter value over a given range (e.g., a correlation coefficient ρ ranging from − 1 to 1) is equally likely, can also be used. However, they can produce Bayes factors that can be biased towards null models, so they are generally not recommended [21]. Combining the prior distribution with the observed data forms the posterior distribution. A Bayes factor is the ratio between the marginal likelihoods of the null model and the alternative model.

**Fig. 1:**
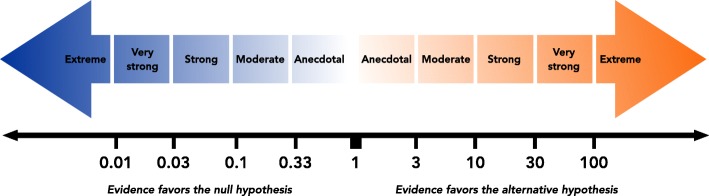
Lee and Wagenmakers’ classification scheme for interpreting Bayes factors (BF_10_). This classification scheme [18, 23], which has been adopted in JASP, provides descriptive labels for interpreting a range of Bayes factors. While this scheme provides a useful starting point for understanding Bayes factor values and may be suitable for many research questions, Bayes factors should be carefully interpreted in light of the research question at hand

Bayesian hypothesis tests in the biobehavioral sciences typically yield Bayes factor values between 0.01 and 100 [22]. Descriptive classification schemes are often used to interpret Bayes factors e.g., [18, 23, 24]. The classification scheme adopted by JASP [23], which is an adaption of Jeffery’s scheme [18], proposes a series of labels for which specific Bayes factor values can be considered “anecdotal”, “moderate”, “strong”, “very strong”, or “extreme” relative evidence for a hypothesis (Fig. 1). Bayes factor classification schemes may facilitate scientific communication [25] as they provide approximate guidelines for Bayes factor interpretation. However, any rigid scheme used to describe Bayes factors cannot be suited to all possible research contexts. For instance, theoretically implausible claims should require more evidence than usual for their support. As we agree that Bayes factors should be interpreted in light of the research context [26] and wish to highlight the direct interpretability of Bayes factors, we do not characterize the results in the present manuscript’s worked examples using an explicit classification scheme. We do, however, mention Lee and Wagenmakers’ classification scheme [23] here given its use in JASP, its relevance in many research contexts, and to provide a preliminary frame of reference for readers that are new to Bayes factors.

Psychiatry researchers are typically concerned with three broad types of research questions: i) How strong is the relationship between continuous variables? ii) are a set of categorical variables interrelated? iii) do groups differ on a continuous explanatory variable, and does this difference covary depending on other variables? While *p*-values are typically used to answer these questions, we will demonstrate that Bayesian inference provides a useful addition to classical hypothesis testing. Bayesian inference is by no means a new concept but its widespread adoption has been hampered, in part, by the inaccessibility of software packages to perform Bayesian analysis. The recent development of the open-source JASP statistical package [25, 27] provides a straightforward means of performing both classical and Bayesian inference using a graphical interface. The aim of this article is to demonstrate that Bayesian hypothesis testing is no more difficult to perform than significance tests, and allows for richer inference than relying exclusively on classical frequentist methods that dominate hypothesis testing in both basic and clinical psychiatry research.

## Methods

A dataset from van Cappellen and colleagues [28] will be used to compare and contrast NHST and Bayesian hypothesis testing using JASP (version 0.8.5.1). A core feature of JASP is the ability to save the entire analysis pipeline as a .jasp file, which includes the data, analysis input options, and output. Thus, interested readers can follow each step of the described analyses by examining the associated .jasp file (https://osf.io/emz4r/).

The primary interest of the study from van Cappellen and colleagues [28] was whether a single intranasal administration of the neuropeptide oxytocin could impact self-reported spirituality. The role of the oxytocin system in human interconnection has been the subject of considerable research interest in psychiatry [29], however, it is not known if the oxytocin system is also involved in spiritual interconnection. In this study, participants were randomized to receive a single administration of either intranasal oxytocin or placebo, after which they responded to measures assessing spirituality. One of the outcomes used to index spirituality was a single item measure that asked, “Right now, would you say that spirituality is important for you?”. After receiving the nasal spray, participants responded on a scale from 0 (Not at all) to 7 (Completely). The study dataset was collected from manuscript’s Open Science Framework webpage (https://osf.io/rk2x7/) For pedagogic purposes, several variables not used in the current demonstration were removed from the original dataset and we perform additional analyses that were not reported in the original manuscript.

## Results

### Correlations

**Fig. 2:**
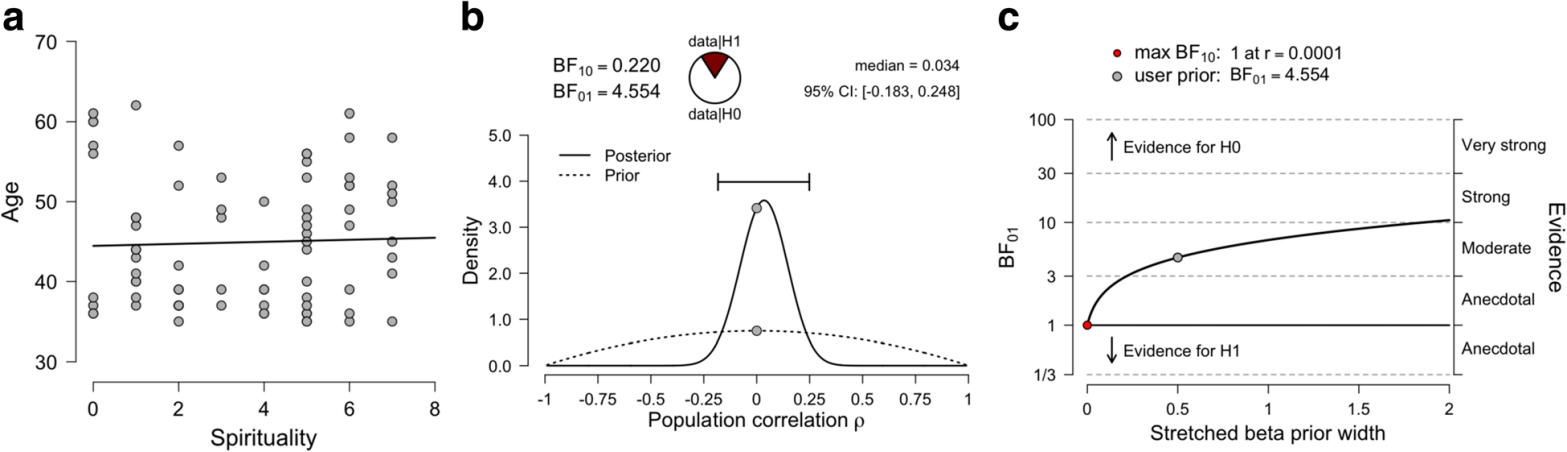
Correlation analysis. A scatterplot visualizing the relationship between age and spirituality (**a**). The prior and posterior distribution for the relationship between age and spirituality (**b**). A robustness check illustrating the effects of assigning a range of stretched prior widths on Bayes factor values (**c**). The grey dot represents the selected prior (stretched beta prior width of 0.5)

Before performing the primary analysis, it is of interest to first assess if spirituality is related to age. A scatterplot visualization of this data suggests that age and spirituality is not related (Fig. 2a). A Pearson correlation coefficient confirms our intuitions, as there is no statistically significant relationship between age and spirituality [Pearson’s *r* = 0.04, 95% CI (− 0.19, 0.26), *p* = 0.75]. Nonetheless, with *p*-values, we cannot be certain if non-significance is due to data insensitivity or to evidence supporting a lack of relationship between these two variables [4, 19, 30].

For our Bayesian analysis, we will compare two models: the null hypothesis (*H*_0_) that the data is distributed according to a bivariate normal distribution with zero covariance — and therefore that there is no correlation between the spirituality and age (i.e., ρ = 0) — and the alternative hypothesis (*H*_1_) that age and spirituality distributed according to a bivariate normal distribution with a non-zero covariance are related. A default prior probability distribution for ρ restricts the parameter space between any value of − 1 and 1, however, values around ρ = 0 are far more likely. We can prescribe more mass to values around ρ = 0 by assigning a smaller stretched beta prior width. Here, we assigned a stretched beta prior [31], with a width of 0.5, in the JASP interface. The dashed line in Fig. 2b illustrates the prior distribution for our example. We now test how the observed data updates our prior distribution with the posterior distribution. Assuming that there is a relationship between age and spirituality, the estimate of the correlation coefficient (ρ) was 0.03 and the central credible interval ranged between − 0.18 and 0.25, which suggests that we are 95% confident that the true value of ρ is located within these bounds. Although confidence intervals were calculated for the NHST analysis described above, these intervals are calculated by average performance over the long run of a series of *future* hypothetical replications. Therefore, it is inaccurate to conclude using NHST confidence intervals that we are 95% confident that the true effect size lies between a set of confidence intervals [2]. However, as the Bayesian framework uses the *present* data to determine the credible interval, then such a conclusion is valid. As BF_01_ = 4.55, this indicates the null model is 4.55 more favored than the alternative model, given the data. Not only does this provide evidence for *H*_0_ relative to *H*_1_ — something not possible with *p*-values — but the Bayes factor also conveys the magnitude of this evidence. Note that JASP reports equivalent BF_10_ and BF_01_ values (Fig. 2b), with the latter simply the inverse of the former. Here, it makes more sense to report the BF_01_ value, as we are more interested in how much more favored the null model (the first subscript number) is than the alternative model (the second subscript number). An illustration of the effects of assigning a range of different prior distributions (i.e., a Bayes factor robustness check) is presented in Fig. 2c. If the data is not bivariate normal, then the Bayesian equivalent to Kendall’s tau [32] is also available as an analysis option in JASP.

### Frequency distributions

Next, we would like to assess if participants could correctly identify whether they had been administered an oxytocin or placebo spray. As several participants responded that they did not know, only definitive “oxytocin” or “placebo” responses were assessed in the original article. Thus, a recoded variable only including definitive responses was added to the present dataset to reproduce the original analysis. As reported in the original article, a classical χ^2^ test suggests that these groups are not distributed differently [χ^2^(1) = 1.55, *p* = 0.21]. The log odds ratio for this analysis was − 0.92 [95% CI (− 2.4, 0.54)]. Like the previous analysis of correlational data, this does not provide any evidence for the null hypothesis nor provide any confidence that the true log odds ratio lies between the CI bounds. Bayesian frequency distribution analysis was performed using independent multinomial sampling, as the crucial test was a comparison of two proportions and the number of people assigned to receive each treatment was presumably fixed [33, 34]. The median log odds ratio was − 0.86, with a 95% credible interval of − 2.31 and 0.51. The null model was only slightly favored over the alternative model (BF_01_ = 1.16). A Bayes factor close to 1 suggests that there were too few data for this analysis [4].

### T-tests

The primary outcome of interest is whether intranasal oxytocin modulates self-reported spirituality. An independent samples Welch’s t-test reveals increased ratings of spirituality after oxytocin (mean = 3.84; SD = 2.26) compared to placebo (mean = 3.25; SD = 2.34), however this was not statistically significant [*t*(75.98) = 1.14, *p* = 0.26, Cohen’s *d* = 0.26, 95% CI for Cohen’s *d* (− 0.19, 0.7)]. If there was a pre-registered directional hypothesis for group differences, then a one-sided t-test, in which the alternative hypothesis is that the oxytocin group would report increased feelings of spirituality compared to the placebo group, would be a valid approach [35]. In this case, however, a one-sided t-test was also not statistically significant (*p* = 0.13).

**Fig. 3:**
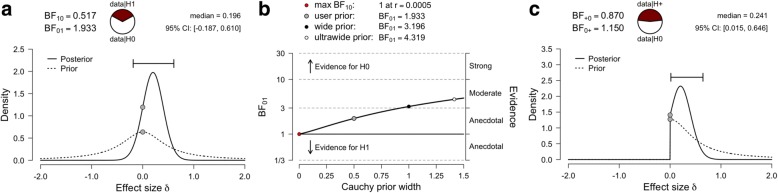
Bayesian analysis of group mean differences. The prior and posterior distribution plot for the analysis of group mean differences (**a**). A robustness check illustrating the effects of assigning wide and ultrawide Cauchy prior widths on Bayes factor values (**b**). The prior and posterior distribution plot for a directional analysis of group differences (**c**). Here, the prior distribution can be set with more mass around zero, but no mass less than zero

For our Bayesian *t*-test alternative, we compare two models for effect size δ: the null hypothesis that the spirituality rating effect sizes for each intervention groups are equal (δ = 0), and the alternative hypothesis that the mean spirituality ratings of each group are different [JASP implements methods described by Rouder and colleagues [19]]. Here, we assign δ a Cauchy distribution prior centered on zero [18], with an interquartile range *r* = 0.5 [δ ∼ Cauchy(0, 0.5)]. A default Cauchy prior with a scale parameter of 0.5 (which presumes we are 50% confident that the true effect size will lie between −.5 and .5) is used, so that the *H*_1_ model includes more realistic effect sizes. The corresponding Bayes factor provides anecdotal evidence for the null hypothesis relative to the alternative hypothesis (BF_01_ = 1.93; Fig. 3a), with a posterior median of 0.2 and a 95% credible interval range of − 0.2 to 0.61. As this BF_01_ value was close to 1, this is suggestive of data insensitivity [4]. In other words, more data needs to be collected. A robustness check was also performed to assess sensitivity to the prior (Fig. 3b), with a wide prior yielding a BF_01_ = 3.2. While some would consider this BF_01_ value as moderate support for the null hypothesis, a wide Cauchy distribution scaling factor of 1 presumes we are 50% confident that the true effect will lie between *d* = − 1 and *d* = 1, which would be unrealistic for most areas of psychiatry. Directional hypothesis testing, similar to a classical one-sided *t*-test, is also possible with a Bayesian framework. Prior distributions can incorporate prior knowledge and be constrained to specific intervals. With a pre-registered hypothesis that intranasal oxytocin can increase ratings of spirituality (*H*_+_), the prior distribution can be set with more mass around zero (as per our non-directional test), but no mass less than zero (Fig. 3c). The directional test provided only very modest support in favor of the *H*_0_ model compared to the *H*_+_ model (BF_01_ = 1.2).

**Fig. 4:**
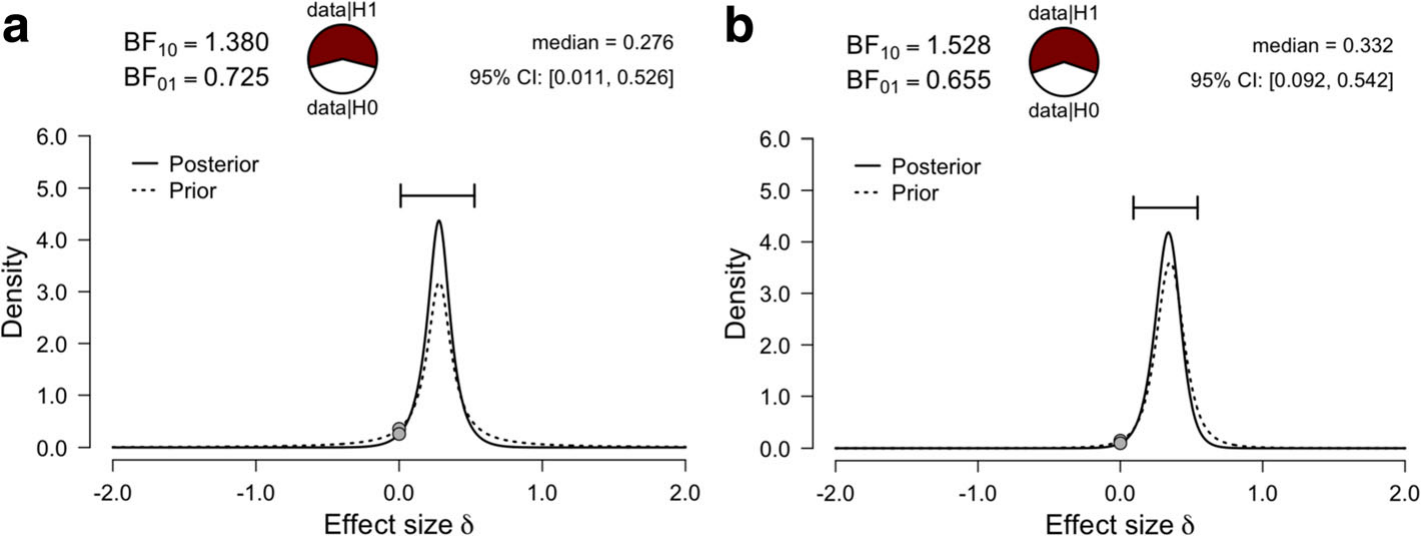
Informed prior distributions. The prior and posterior distribution plot for the analysis of group mean differences using a prior informed by existing knowledge of the average effect size reported in oxytocin studies (**a**). The prior and posterior distribution plot for the analysis of group mean differences using an informed “Oosterwijk prior”, which represents small-to-medium effect sizes (**b**)

A final consideration for Bayesian *t*-tests is that that the default prior distribution centered on zero may not best represent prior expectations of an effect size, or the small-to-medium effect sizes commonly observed in the biobehavioral sciences. Instead of using a default prior distribution, an informed distribution can nominate the central location and scale of a prior distribution. For example, we can use the average effect size of oxytocin studies in healthy individuals of *d* = 0.28 [36] as the central location with a Cauchy scale of 0.1, which might be considered a more realistic prior distribution. The corresponding Bayes factor provides only very modest evidence for the alternative hypothesis (BF_10_ = 1.38; Fig. 4a), with a posterior median of 0.28 and a 95% credible interval range of 0.01 to 0.53. Although we now have evidence for alternative hypothesis relative to the null hypothesis when using an informed prior (as opposed to evidence for the null model when using a default prior), this evidence is still quite weak. Without explicit prior information, the “Oosterwijk prior” (a t-distribution centered at 0.35, with a scale of 0.102 and 3 degrees of freedom) can be used as an informed prior, which is representative of the small-to-medium effects commonly observed in the biobehavioral sciences [37]. The informed Oosterwijk prior yielded a BF_10_ of 1.53 (Fig. 4b; posterior median of 0.33; 95% credible interval range of 0.09 to 0.54), which was a similar result to the first informed prior we presented.

### ANCOVA

Given the potential effect of religious affiliation, we will now carry out an ANCOVA on the main effect of nasal spray administration condition with religious affiliation entered as a covariate, which was the approach used by van Cappellen and colleagues [28]. The categorical variable for religious affiliation was recoded in the present dataset to recreate the NHST results from the original study (i.e., the original variable, with seven religious affiliation categories, was recoded into a “yes” or “no” response for whether participants were religiously affiliated). As previously reported, when explaining away the error variance attributable to religious affiliation (commonly referred to as “controlling” for a covariate), oxytocin increases spirituality (F(1, 75) = 4.87, *p* = 0.03, η_p_^2^ = 0.061). For the Bayesian ANCOVA [38], a model including intervention group and religious affiliation will be compared against the null model, which only contains religious affiliation (See Table 1 for included models). The default JASP multivariate Cauchy priors (fixed effects Cauchy prior scale parameter for fixed effects = 0.5, Cauchy prior scale parameter for covariates = 0.354) will be used, although these parameters can be adjusted. As Bayes factors have a transitive relationship [39], the model with intervention group and religious affiliation (BF = 398,231) can be compared to the religious affiliation model (BF = 230,440) by division (398,231/230440 ≃ 1.73). Thus, after explaining for the error variance attributable to religious affiliation, oxytocin increases spirituality. However, as the oxytocin condition + religious affiliation model was only preferred to the oxytocin model by a factor of 1.73, this could be considered only very modest evidence. Given the modest magnitude of this Bayes factor, this does not suggest that there was no effect, but rather that the observed data were insensitive to detect an effect (i.e., more participants might be required). This is consistent with recent concerns surrounding statistically underpowered oxytocin studies [36].

**Table 1:** Bayesian ANCOVA models

Model type	Model contents	BF_10_	BF_01_
Null model	Only participants have effects	1	1
Condition model	Null model + main effect of condition	0.41	2.44
Religious affiliation model	Null model + main effect of religious affiliation	230,440	< 0.001
Condition + religious affiliation model	Null model + condition + religious affiliation	398,231	< 0.001

### ANOVA

The final analytical approach to be presented is repeated measures ANOVA, which will be used to assess the main effects of time and nasal spray condition on spirituality ratings, and the interaction of time and nasal spray condition. This analysis reveals no significant main effect of time (F(1, 74) = 0.21, *p* = 0.65, η_p_^2^ <  0.01), treatment (F(1, 74) = 1.25, *p* = 0.27, η_p_^2^ = 0.02), or time × treatment interaction (F(1, 74) = 0.08, *p* = 0.78, η_p_^2^ <  0.01). A Bayesian repeated measures ANOVA compares a series of different models against a null model [40]. We will compare 4 models against the null model (Table 2). Of note, the interaction model also includes the main effects model, as interactions without corresponding main effects are considered implausible [41]. The default JASP prior for fixed effects will be used (r scale prior width = 0.5). Here, the null model was 7.85 times more favored than the main effects model and 32.21 times more favored than the interaction model (Table 2). There was moderate evidence that the null model was more favored than the time model (BF = 5.34), but only very little evidence it was more favored than the condition model (BF = 1.54), which is suggestive of insensitive data. Comparison of the main effects model with the interaction model (7.85/32.21) reveals that the main effects model was preferred to the interaction model by a BF of 4.17 (i.e., 1/0.24).

**Table 2:** Bayesian ANOVA models

Model type	Model contents	BF_10_	BF_01_
Null model	Only participants have effects	1	1
Time model	Null model + main effect of time	0.19	5.34
Condition model	Null model + main effect of condition	0.65	1.54
Main effects model	Null model + time model + condition model	0.13	7.85
Interaction model	Main effects model + interaction effects	0.03	32.21

## Conclusions

A comparison of classical and Bayesian inferential frameworks reveals that the Bayesian approach can complement *p*-values and effect sizes by providing additional information for hypothesis testing (Table 3). Not only do Bayes factors quantify relative evidence for both *H*_1_ and *H*_0_, the magnitude of this evidence is also presented as an easy-to-interpret odds ratio. For demonstration, we have provided worked examples of Bayesian analysis for common statistical tests in psychiatry using JASP. Interested readers that would like to perform other types of Bayesian analysis not currently available in JASP, or require greater flexibility with setting prior distributions can use the ‘BayesFactor’ R package [42].

**Table 3:** A comparison of NHST and Bayesian inference

Test	NHST	Bayes
Correlation	No significant relationship (*p* = 0.75)	Null model 4.55 times more favored than the alternative model
Chi-squared test	No significant difference (*p* = 0.21)	Null model 1.16 times more favored than the alternative model
T-test	No significant difference (*p* = 0.26)	Null model 1.93 times more favored than the alternative model
ANCOVA	Significance difference (*p* = 0.03)	Covariate model 1.73 times more favored than oxytocin model
ANOVA - time effect	No main effect (*p* = 0.65)	Null model 5.34 times more favored than time model
ANOVA - condition effect	No main effect (*p* = 0.27)	Null model 1.54 times more favored than oxytocin model
ANOVA - time*condition	No interaction effect (*p* = 0.78)	Main effects model 4.17 times more favored than interaction model

A few limitations should be considered to help ensure Bayes factors are used appropriately. First, if researchers wish to present the size of an effect then the presentation of an effect size and corresponding confidence (or credible) interval is important, as Bayes factors alone can only present the support of the alternative hypothesis model against a null model. Second, changing the width of the prior will also change the Bayes factor — sometimes substantially so. But this is not necessarily a limitation, as robustness checks can be used ensure the evidence is robust to different prior specifications [43]. Thus, we recommend reporting all assumptions that the results depend on, along with robustness checks. Third, inference from Bayes factors depends on the models being compared. One could compare a non-null hypothesis (e.g., small effect *δ* = 0.05) to the alternative prior distribution. This may provide similar evidence for a small effect compared to the alternative as comparing the null (*δ* = 0) to the same alternative. Thus, Bayes factors should never be interpreted in absolute terms as providing evidence for or against the null hypothesis. As a consequence, it is inaccurate to say that a Bayes factor can “prove the null”, as we are only assessing evidence for a null model proportional to an alternative model. Fourth, when computing Bayes factors, the prior is often suggested to quantify our belief about the parameters in question, or to represent our hypothesis. However, in practice, using default prior distributions does not express *question specific* information, unless we believe all parameters are the same or these defaults happen to suit our hypotheses. Keeping this caveat in mind, we have largely adopted the default prior approach, which is generally advocated for within the psychological literature [44]. However, for comparison we also present an informed prior approach for *t-*tests.

Altogether, Bayesian statistics adds an additional family of procedures to the researcher’s statistical toolkit, which can be used to complement classical frequentist statistics. To help facilitate the wider adoption of Bayesian statistics, we recommend that researchers present Bayes factors alongside *p*-values and effect sizes, with corresponding confidence intervals. We also encourage researchers to accompany their manuscripts with corresponding .jasp files. As .jasp files integrate data, analysis input options, and output this will allow readers to inspect and recreate reported analyses, which is an important pillar of reproducible science [45].

## Abbreviations

NHST: Null hypothesis significance testing
